# Rational construction of a high-quality and high-efficiency biosynthetic system and fermentation optimization for A82846B based on combinatorial strategies in *Amycolatopsis orientalis*

**DOI:** 10.1186/s12934-024-02464-4

**Published:** 2024-06-28

**Authors:** Xinyi Zhao, Chenyang Zhu, Wenli Gao, Huang Xie, Zhongyuan Lyu, Qingwei Zhao, Yongquan Li

**Affiliations:** 1 First Affiliated Hospital and Institute of Pharmaceutical Biotechnology, Zhejiang University School of Medicine, Hangzhou, 310058 China; 2 Zhejiang Provincial Key Lab for Microbial Biochemistry and Metabolic Engineering, Hangzhou, 310058 China; 3 Institute of Biopharmaceuticals, School of Pharmaceutical Sciences, Taizhou University, Taizhou, 318000 China

**Keywords:** Oritavancin, A82846B, Biosynthetic system, Strain improvement, Fermentation optimization, Combinatorial strategies

## Abstract

**Background:**

Oritavancin is a new generation of semi-synthetic glycopeptide antibiotics against Gram-positive bacteria, which served as the first and only antibiotic with a single-dose therapeutic regimen to treat ABSSSI. A naturally occurring glycopeptide A82846B is the direct precursor of oritavancin. However, its application has been hampered by low yields and homologous impurities. This study established a multi-step combinatorial strategy to rationally construct a high-quality and high-efficiency biosynthesis system for A82846B and systematically optimize its fermentation process to break through the bottleneck of microbial fermentation production.

**Results:**

Firstly, based on the genome sequencing and analysis, we deleted putative competitive pathways and constructed a better A82846B-producing strain with a cleaner metabolic background, increasing A82846B production from 92 to 174 mg/L. Subsequently, the PhiC31 integrase system was introduced based on the CRISPR-Cas12a system. Then, the fermentation level of A82846B was improved to 226 mg/L by over-expressing the pathway-specific regulator StrR via the constructed PhiC31 system. Furthermore, overexpressing glycosyl-synthesis gene *evaE* enhanced the production to 332 mg/L due to the great conversion of the intermediate to target product. Finally, the scale-up production of A82846B reached 725 mg/L in a 15 L fermenter under fermentation optimization, which is the highest reported yield of A82846B without the generation of homologous impurities.

**Conclusion:**

Under approaches including blocking competitive pathways, inserting site-specific recombination system, overexpressing regulator, overexpressing glycosyl-synthesis gene and optimizing fermentation process, a multi-step combinatorial strategy for the high-level production of A82846B was developed, constructing a high-producing strain AO-6. The combinatorial strategies employed here can be widely applied to improve the fermentation level of other microbial secondary metabolites, providing a reference for constructing an efficient microbial cell factory for high-value natural products.

**Supplementary Information:**

The online version contains supplementary material available at 10.1186/s12934-024-02464-4.

## Introduction

Microbial-derived drugs play a pivotal role in medical and healthcare fields, including anti-infection, antitumor, immunosuppression and metabolic regulation. In the pharmaceutical industry, synthesizing structurally complex compounds derived from microorganisms through chemical methods is often challenging and not easily scalable. Alternatively, utilizing microbial cell factories for biosynthesis is an efficient and cost-effective method for the large-scale production of target products. However, efficient and environmentally friendly production still faces formidable challenges such as low production efficiency and difficulties in separation. Therefore, genetic modification of microbial biosynthetic systems coupled with fermentation optimization is pivotal in enhancing pharmaceutical production based on the advancement of synthetic biology and genomics.

The increasingly concerning issue of drug resistance in Gram-positive pathogens has led to an urgent need for the development of antibiotics to deal with it. Oritavancin is a new generation of semi-synthetic glycopeptide antibiotics against Gram-positive bacteria, which is used to treat acute bacterial skin and skin structure infections (ABSSSI) [1]. It plays the bactericidal role by blocking the transglycosylation of peptidoglycan biosynthesis, thus inhibiting the formation of bacterial cell walls and resulting in the death of bacterial cells [2]. Notably, oritavancin is the only antibiotic with a single-dose therapeutic regimen for ABSSSI, allowing patients to complete a course of treatment with a single injection based on the advantage of prolonged half-life, convenient administration, and mild adverse reactions [3]. Additionally, it exhibits potent antimicrobial activity against vancomycin-resistant/vancomycin-insensitive *staphylococci* and *enterococci* species, thus the application of oritavancin can effectively reduce the development of resistance [4].

The glycopeptide antibiotic A82846B is the key precursor of oritavancin, which is produced by *Kibdelosporangium aridum* and *Amycolatopsis orientalis* through microbe fermentation [4–6]. The major processes involved in the biosynthesis of A82846B are as follows: (i) synthesis of non-protein amino acids; (ii) synthesis of the heptapeptide backbone by non-ribosomal peptide synthetase (NRPS) assembly line; (iii) post-modification of the heptapeptide backbone (chlorination, oxidative cross-linking, methylation, glycosylation) [7–9]. During the fermentation of A82846B, another two homologous impurities (A82846A and A82846C) are produced as well in natural producers, which differ only in the number of chlorine substituents: A82846B has two chlorines in the side chain of amino acid residues at positions 2 and 6 respectively, A82846A has only one chlorine substitution at position 2, but there is no chlorine substitution in A82846C [10]. The structural similarity of these three compounds resulted in the difficulty of isolation and purification, thus increasing the commercialization cost of oritavancin.

At present, studies on oritavancin mainly focus on the antibacterial mechanism, clinical application, pharmacokinetics, and other applications, while the synthesis of its direct precursor A82846B is rarely studied [11–14]. In recent years, genetic engineering and various physicochemical mutagenesis have been used to increase the yield of A8284B and reduce the proportion of impurities. In 2018, Wang et al. overexpressed the *chal* gene encoding the halogenase in A82846B gene cluster of *Amycolatopsis orientalis* SIPI18099 to reduce the production of homologous impurities A82846A and A82846C, but they cannot be eliminated. The yield of A82846B in the recombinant strain *A. orientalis* chal-3 reached 2200 mg/L with the generation of 290 mg/L A82846A and 56 mg/L A82846C in a 5 L fermenter [6]. In 2020, another study reported that the strain *Kibdelosporangium aridum* SIPI-3927-C6 was constructed by integrating *orf10-*encoded halogenase and *orf11*-encoded glycosyltransferase A, of which the A82846B production was 2520 mg/L but with 13.4% A82846A and 0.1% A82846C in a 5 L fermenter [5]. In these studies, although the fermentation level of A82846B was enhanced, the impact of impurities on the cost of separation and purification as well as industrial application has not been eliminated. In our reported work, we used the vancomycin producer *Amycolatopsis orientalis* AO-1 as a chassis to construct the A82846B synthetic pathway by replacing the glycosyl-transferases module (*gtfDE*) and glycosyl-synthesis module (*vcaAEBD*) in vancomycin gene cluster with the corresponding modules (*gtfABC* and *evaAEBD*) in A82846B cluster respectively. *Amycolatopsis orientalis* AO-2 for producing A82846B was successfully constructed without generating homologous impurity, but its fermentation level was very low (89 mg/L) [15]. Based on this, we would like to use AO-2 as the original strain to achieve the high-quality and high-efficiency biosynthesis of A82846B.

**Fig. 1:**
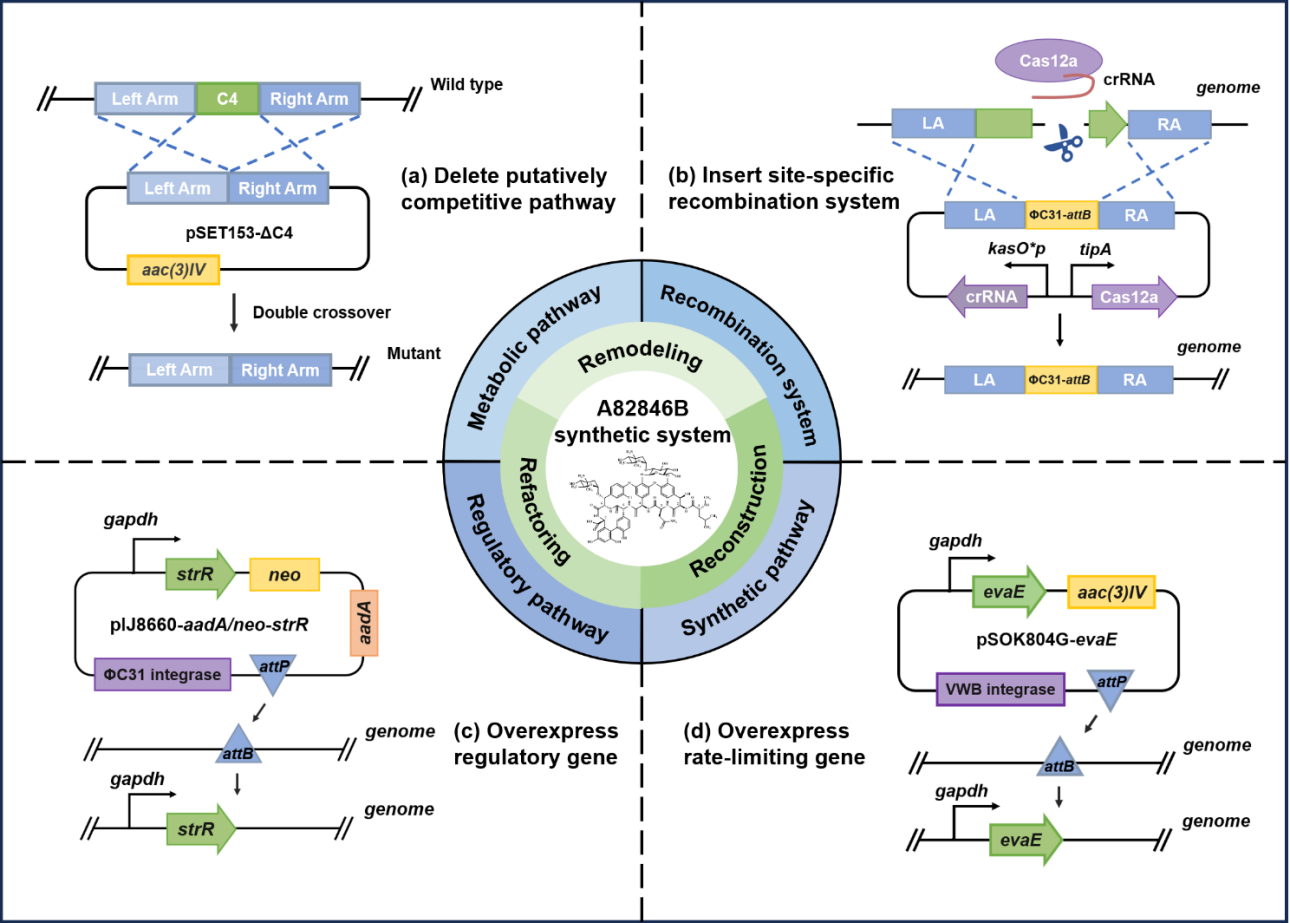
Schematic representation of constructing a high-quality and high-efficiency biosynthetic system of A82846B

In this study, we applied a stepwise strategy of combining genome remodeling, regulatory network refactoring and synthetic pathway reconstruction to rationally construct a high-quality and high-efficiency biosynthetic system of oritavancin direct precursor A82846B via CRISPR gene editing technology and other means (Fig. 1). Moreover, the fermentation medium and conditions were optimized to lay the foundation for scale-up production, and the potential of A82846B production was also evaluated in a 15-L fermenter based on further fermentation optimization. The results of this study provided ideas for improving the biosynthetic system of antibiotics, which is of guiding significance for constructing high-yielding strains of microbial natural products. Combinatorial strategies used in this work are generally applicable to other industrial strains.

## Materials and methods

### Strains, plasmids, and primers

Strains and plasmids used in this study are listed in Additional file: Table S1; primers are listed in Additional file: Table S2.

### Construction of plasmids

For deletion of several core genes in cluster C4, two 2.5-kb DNA fragments flanking the region were amplified from the genomic DNA of *A. orientalis* AO-2 using primer pairs 7 and 8 respectively, and then cloned into *Hin*dIII-digested pSET153 generating the disruption plasmid pSET153-ΔC4.

The coding sequence of PhiC31-*attB* was synthesized according to the reported literature [16], and amplified using primer pair 10. Two 2.5-kb DNA fragments of homologous arms were amplified with primer pairs 11 and 12 respectively. These fragments were cloned into *Spe*I/*Nhe*I-digested pKCCas12a by ClonExpress MultiS One Step Cloning Kit (Vazyme Biotech, Nanjing, China). Subsequently, the crRNA array expression cassette containing the spacer targeting PhiC31-*attB* insertion area was amplified with primer pair 13 and inserted into the *Nde*I/*Spe*I site to construct the plasmid pKCCas12a-PhiC31.

The fragment of reporter gene *gusA* was synthesized and then cloned into *Bgl*II/*Nde*I-digested pIJ8660-*aadA/neo* to construct the integrative reporter vector pIJ8660-*aadA/neo-gusA.* The regulator *strR* was obtained by PCR amplification with primer pair 15 and then cloned into *Bgl*II/*Nde*I-digested pIJ8660-*aadA/neo* for gene overexpression.

To construct integrative plasmids pSOK804G-*gene*, glycosyl-synthesis genes and glycosyl-transfer genes were amplified by PCR with primer pairs 16 to 24 from the genomic DNA respectively, and then these fragments were individually cloned into the *Kpn*I/*Eco*RI site of pSOK804G. pSOK804G was derived from pSOK804 by inserting the fragment of strong promoter *gapdh.*

### Construction of *A. orientalis* strains

All of the constructed plasmids were transformed into *E. coli* ET12567/pUZ8002 and then introduced into *A. orientalis* via intergeneric conjugation.

For gene deletion, the plasmid derived from pSET153 was introduced into *A. orientalis* AO-2 as mentioned above. The conjugants were cultivated on YMG plates supplemented with 200 µg/mL apramycin at 30 °C to select single-crossover-recombination strains. Subsequently, after two rounds of growth on plates without antibiotics, double-crossover mutants were selected by apramycin sensitivity and verified by PCR.

For PhiC31 system insertion, the mutant strain was constructed by using the tipACas12a system. The transformants were selected on a solid YMG medium containing 200 µg/mL apramycin for further confirmation by PCR, and the resulting strains were cultivated with 5 µg/mL thiostrepton induction. These strains subsequently were grown on YMG plates without apramycin at 42 °C for two rounds to remove the CRISPR-Cas12a plasmid. The mutant strain was identified by PCR and DNA sequencing.

To confirm the integration of *strR* overexpression plasmid into the genome, the transformants were selected on a YMG medium with 50 µg/mL kanamycin and confirmed by PCR. The overexpression strains of glycosyl-synthesis modules and glycosyl-transfer modules were selected by culturing exconjugants on YMG agar plates supplemented with 200 µg/mL apramycin and identified by PCR.

### Culture conditions

*E. coli* and its derivatives were grown in Luria − Bertani medium (1% tryptone, 0.5% yeast extract, and 1% NaCl) at 37 °C. The solid media for *A. orientalis* strains were as follows: YMG medium (0.4% yeast extract, 1.0% malt extract, 0.4% glucose, 0.2% CaCO_3_, 2% agar), MS medium (2% soybean powder, 2% mannitol, 2% agar). The fermentation media used for *A. orientalis* were as follows: TSB medium (2% tryptone, 0.5% NaCl, 0.25% glucose, 0.25% K_2_HPO_4_), GP medium (glucose-peptone medium; 2% glucose, 0.5% peptone, 0.1% NaCl, 0.05% KCl, 0.08% MgSO_4_, and 0.01% KH_2_PO_4_), GPM medium (glucose-peptone-maltodextrin medium; 2.2% glucose, 0.6% peptone, 1.2% maltodextrin, 0.1% NaCl, 0.05% KCl, 0.08% MgSO_4_, and 0.01% KH_2_PO_4_).

### Optimization of fermentation medium and conditions

The Plackett-Burman design (PBD) was used to evaluate the main effect factors of medium composition and the Box-Behnken design (BBD) of response surface methodology (RSM) was applied to analyze the most optimum level and interactions of the screened significant factors. The PBD and BBD were performed using Design-Expert 10.0.7 software. Single-factor experiments were carried out to evaluate the optimal fermentation conditions at the shake flask level.

### Fermentation of *A. Orientalis* and HPLC analysis

The strains were cultured on YMG agar plates for about 5–7 days at 30 °C for growth and then inoculated into 20 mL TSB medium as seed medium. After incubation at 30 °C and 220 rpm for 48 h, 1.0 mL of seed culture was inoculated into 30 mL fermentation medium in 250 mL flasks and then cultured at 30 °C and 220 rpm for 144 h.

For analysis of A82846B, the culture sample was extracted with an equal volume of methanol and then the supernatant was recovered by centrifugation at 12,000 rpm for 10 min. After filtering through a 0.22 μm membrane, the supernatant was injected into the Agilent 1260 HPLC system, equipped with a ZORBAX Eclipse XDB C18 250 mm column, following the program with mobile phases A (0.1% formic acid in distilled water) and mobile phases B (acetonitrile): 0→15 min, A: B = 95:5→85:15; 15→20 min, A: B = 85:15→0:100; 20→25 min, A: B = 0:100→95:5, 25→30 min, A: B = 95:5. The flow rate was 1 mL/min and the UV detection was set at 280 nm.

### Fermentation of *A. Orientalis* AO-6 in a 15 L stirred-tank bioreactor

Scale-up production of A82846B was conducted in a 15 L stirred-tank bioreactor (Biotech-15JS, China) with a working volume of 10 L of culture medium, containing antifoam (1‰). The culture temperature was maintained at 30℃, and the initial pH was adjusted to 6.5. Dissolved oxygen (DO) was maintained at 25–45% through control of the airflow with 0.5–1.5 vvm (volume per culture volume per minute), and the agitation was 200–600 rpm. Moreover, bioreactor pressure was maintained at 0.04 MPa during the fermentation process. Samples were taken every 24 h for analysis of A82846B concentration.

Based on the results of medium optimization at the shake flask level, the culture medium was further adjusted to meet the demand of scale-up fermentation, as follows: 2.5% glucose, 1.8% peptone, 5% maltodextrin, 0.1% NaCl, 0.05% KCl, 0.08% MgSO_4_, 0.01% KH_2_PO_4_, 0.3% CaCO_3_. This fermentation medium was named GPM-2.

### RNA extraction and quantitative real‑time PCR (qRT‑PCR)

The RNA of *A. orientalis* strains was extracted from mycelia cultured in GP medium for 48 h, using an EASYspin Plus bacteria RNA extract kit (Aidlab Biotech, Beijing, China) according to the manufacturer’s instructions. Residual genomic DNA was digested with gDNA Remover Mix (ABclonal, Wuhan, China). The cDNA was synthesized using ABScript III Reverse Transcriptase (ABclonal, Wuhan, China) according to the manufacturer’s instructions.

To analyze the transcription level of A82846B gene cluster, quantitative real-time polymerase chain reaction (qRT-PCR) was performed using Universal SYBR Green Fast qPCR Mix (ABclonal, Wuhan, China) with the primer pairs described in the Additional file: Table S1. The sigma factor gene *hrdB* was used as an internal control to normalize the transcriptional levels. The fold changes of the transcriptional levels were calculated by the 2^−ΔΔCt^ method [17].

## Results and discussion

### Deletion of putatively competitive gene cluster

Actinomycetes harbor a diverse and abundant source of natural products, which possess a genome enriched with gene clusters responsible for natural product synthesis [18, 19]. In addition to the biosynthetic pathways of target secondary metabolites, actinomycetes also boast a range of other secondary metabolic pathways [20, 21]. These secondary metabolic pathways may impede the synthesis of target products by affecting strain growth or competing for specific precursors, so blocking these potentially competitive metabolic pathways is an effective method for enhancing the fermentation level of the desired metabolites [22].

**Fig. 2:**
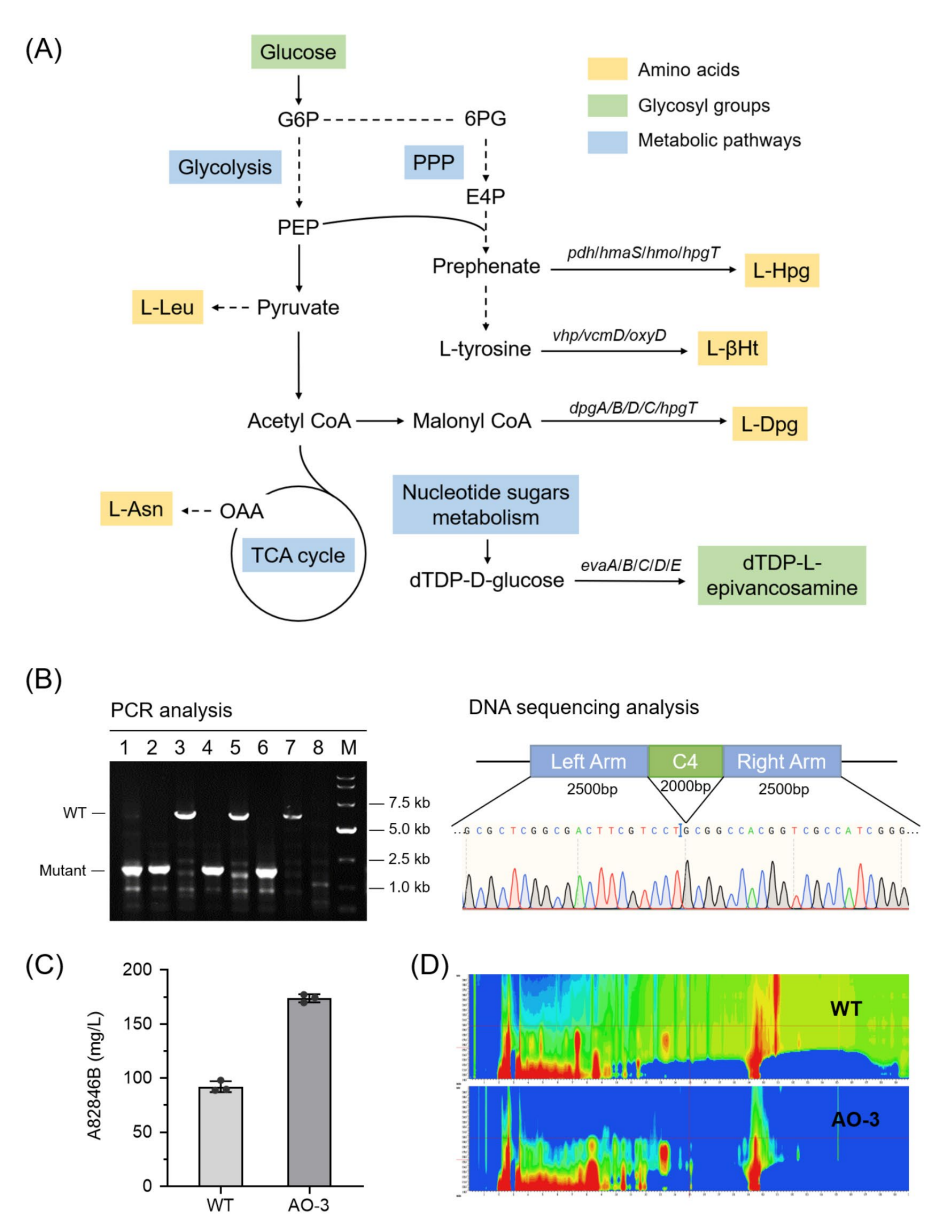
Improvement of A82846B production by deleting putatively competitive gene cluster. **(A)** Schematic representation of metabolic pathways involved in A82846B biosynthesis. Precursors for A82846B biosynthesis were shown in yellow block (five amino acids) and green block (glycosyl groups). Metabolic pathways were shown in the blue block. Some important genes involved in the biosynthesis of relative precursors were also shown (italics). PPP, pentose phosphate pathway; G6P, glucose-6-phosphate; 6PG, 6-phosphogluconate; PEP, phosphoenolpyruvate; E4P, erythrose 4-phosphate; OAA, oxaloacetic acid. **(B)** Analysis of the deletion mutant by PCR and DNA sequencing. M represents the DL15000 DNA marker. **(C)** Effect on A82846B production by deletion of key genes in C4. The values shown are the average of three determinations and error bars denote the standard deviation of the means. **(D)** Analysis of metabolite profiles in WT and AO-3 by full wavelength scanning

During the process of A82846B biosynthesis, there are five amino acids (Leu/ βHt/ Asn/ Hpg/ Dpg) required for the NRPS assembly line to generate the heptapeptide backbone (vancomycin aglycone). Subsequently, one D-glucose and two L-epivancosamines are attached to the crosslinked heptapeptide backbone. These amino acids and glycosyl groups derive from multiple fundamentally important metabolic pathways and metabolic intermediates (Fig. 2A). Hence, to reduce the utilization of common precursors by potential competitive pathways, we focused primarily on putative competitive NRPS or NRPS-PKS gene clusters that may compete for some precursors such as acyl, amino acids and glucose.

The secondary metabolite biosynthesis gene clusters in *A. orientalis* AO-2 were analyzed with the anti-SMASH database tool [23]. The results demonstrated that there are twenty-eight putative biosynthetic gene clusters in the genome of AO-2, seven of which are NRPS/ NRPS-PKS gene clusters (Table 1). Except for the gene cluster of target product A82846B, the results of RT-PCR showed that four of the six NRPS/NRPS-PKS clusters were transcribed. Based on the transcriptome gene expression analysis of AO-2, the core gene expression of cluster 4 had the highest level (FPKM = 73 ± 7) among four transcribed NRPS/NRPS-PKS clusters, although it was lower than that of target A82846B gene cluster (FPKM = 244 ± 16) (see Additional file: Fig. S1). Further analysis of cluster 4 revealed that it encodes a compound called ECO-0501. The substrate for its synthetic initiation is acetyl-coenzyme A, which is a pivotal intermediate for the metabolism of energy and matter, and also plays a crucial role in the biosynthesis of A82846B. Additionally, the modification system for ECO-0501 encoded by cluster 4 uses arginine, glycine, and glucose as substrates. Based on these analyses, we hypothesized that there might be potential competitive pathways between the metabolic networks of the A82846B gene cluster and cluster 4.

**Table 1:** Putative NRPS/NRPS-PKS gene clusters predicted in *A. orientalis* AO-2

Cluster	Type	Length (bp)	Transcription
C4	NRPS, T1PKS	144,268	Yes
C5	NRPS, T1PKS	55,561	No
C7	NRPS, NRP-metallophore	58,781	No
C9	NRPS, NRP-metallophore	63,601	Yes
C12	NRPS	52,512	Yes
C13	NRPS, T1PKS	106,391	Yes
C27	A82846B gene cluster	94,993	Yes

We deleted the HTH domain of *orf4* (transcriptional regulator) and *orf5* (responsible for 4-guanidinobutyryl group biosynthesis) in cluster 4, and the resulting mutant was named AO-3. The A82846B production of strain AO-3 increased from 92 mg/L to 174 mg/L after 144 h fermentation, which was 89% higher than the wild-type strain AO-2 (Fig. 2C). Furthermore, by full-wavelength scanning, we found that the resulting strain AO-3 had a much cleaner metabolite profile than AO-2 (Fig. 2D). Additionally, there was no significant difference in physiological parameters such as cell growth, colony morphology and colour of the strains after knocking out the competitive gene cluster (see Additional file: Fig. S2).

We collected fermentation broth from the wild-type strain (AO-2) and mutant strain (AO-3) fermented for 48 h and extracted bacterial RNA. qPCR experiments were performed on the A82846B gene cluster to investigate the transcription level changes of target biosynthetic genes after deleting the hypothetical competing cluster. The results showed that the transcription level of A82846B biosynthesis genes was increased overall, especially the genes related to amino acid synthesis and glycosylation modification (see Additional file: Fig. S1). However, the reasons for these changes in transcription level are still unclear and require more research. Additionally, the specific competitive mechanisms between the target gene cluster and putatively competitive gene cluster can be further explored using some omics tools such as metabolomics and transcriptomics, which would give a better understanding of why target product yields were enhanced.

### Introduction of PhiC31-based recombination system

Site-specific recombination relies on the association of a small range of homologous sequences and requires site-specific protein to catalyze the process. The phage recognizes the *attB* site in the host genome and forms a consortium complex with the *attP* site through its encoded integrase, thereby undergoing strand exchange to integrate the phage genome into the host chromosome [24, 25]. In recent years, site-specific recombinase-mediated integration system has provided a good method for genetic manipulation and been widely used in the field of microbial synthetic biology [26–28].

In the genome of A82846B-producing strain *A. orientalis*, there is only one specific VWB-*att* site, which cannot be used to efficiently express multiple genes. The lack of site-specific recombination systems has posed difficulties for genetic manipulation in the A82846B-producing strain. PhiC31 integrase is an attractive recombinase to promote the integration of the phage DNA sequences into the bacterial genomes by site-specific recombination [29, 30]. The PhiC31 integrase-mediated recombination system has become a commonly used integration tool in the genetic manipulation of actinomycetes. In this paper, we attempted to introduce PhiC31 system into the A82846B-producing strain to improve its integration efficiency and simplify genetic manipulation.

**Fig. 3:**
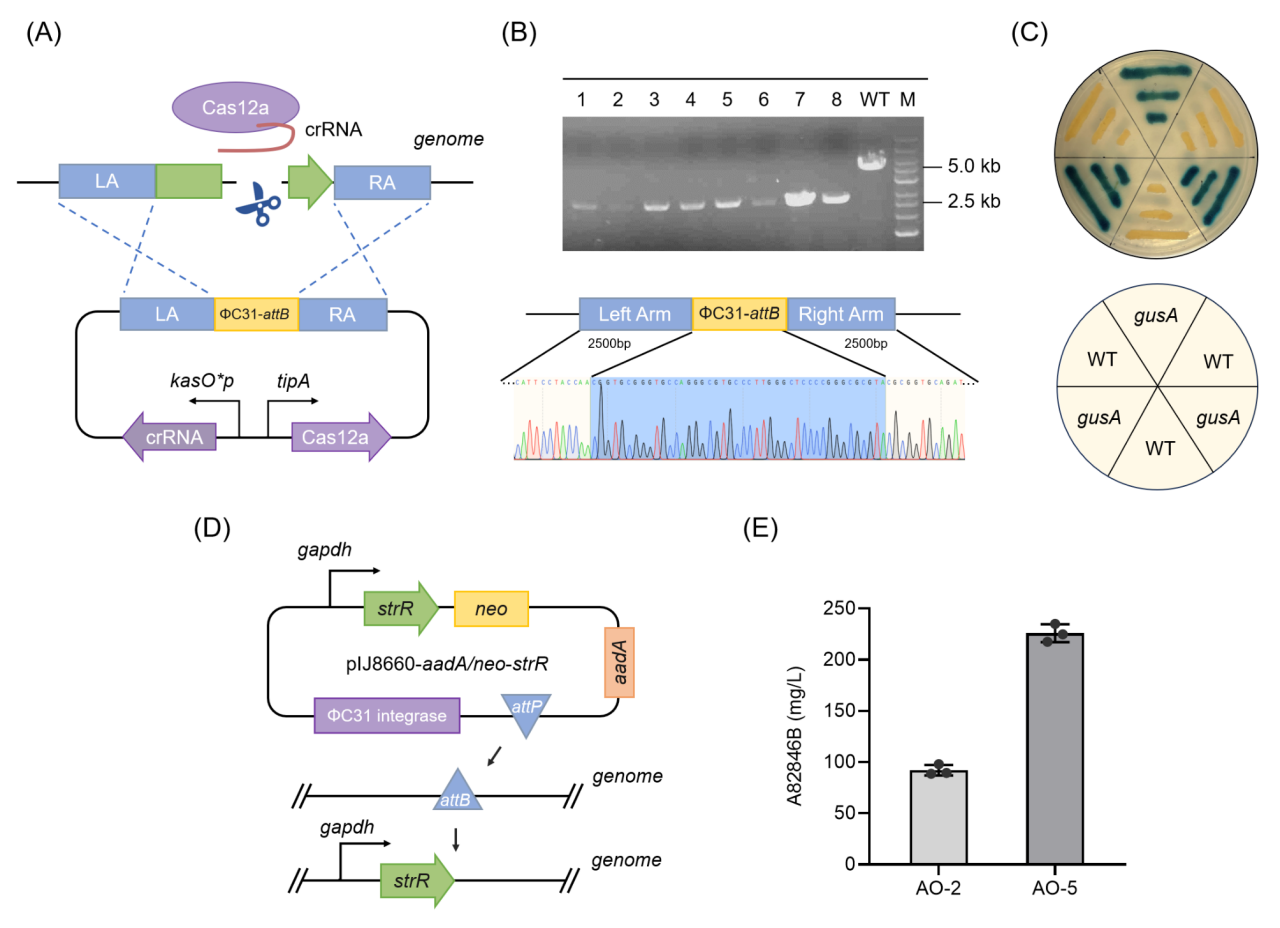
Construction of PhiC31-*attB* site in *A. orientalis* and duplication of regulator for improving A82846B production. **(A)** The scheme of constructing PhiC31-*attB* site based on the CRISPR-Cas12a system. The plasmid contains Cas12a nuclease and crRNA expressed under *tipA* promoter and kasO*p promoter respectively. The 2.5 kb upstream/downstream homologous regions flank the target gene. **(B)** Analysis of the *attB* site mutant strain by PCR and DNA sequencing. M and WT represent the 1 kb DNA ladder and the wild-type strain respectively. **(C)** Evaluation of the effectiveness of artificial PhiC31-*attB* site via the GusA reporter system. The recombinant strain AO-4-*gusA* and the wild-type strain were cultured on YMG agar plates overlaid with an X-Gluc solution. Translated GusA enzyme catalyzes the conversion of the X-Gluc chromogenic substrate, yielding blue-coloured colonies. **(D)** The scheme of *strR* gene overexpression via the PhiC31 integrase-mediated recombination system. *StrR* gene was expressed under strong promoter *gapdh* and Kanamycin resistance gene was used as a screening marker. **(E)** Effect on A82846B production by overexpressing *strR*

The PhiC31-*attB* coding sequence was inserted into the genome of *A. orientalis* AO-3 through the CRISPR-Cas12a system (Fig. 3A). PCR and sequencing analysis confirmed that the mutant strain AO-4 with the PhiC31-*attB* site was successfully constructed (Fig. 3B). To evaluate the effectiveness of the artificial PhiC31-*attB* site via the GusA reporter system, the pIJ8660-*aadA/neo-gusA* plasmid was introduced into the engineered strain AO-4. The recombinant strain AO-4-*gusA* and the wild-type strain were cultured on YMG agar plates overlaid with an X-Gluc solution. As shown in Fig. 3C, it was clear to observe blue colonies of the strain AO-4-*gusA*, indicating that the *gusA* gene was successfully integrated into the genome via the constructed PhiC31-*attB* site and expressed GusA, which catalyzed X-Gluc to generate the blue products.

These results indicated that the PhiC31 integrase system was successfully introduced into the A82846B-producing strain based on the CRISPR-Cas12a system and it could efficiently integrate the target gene into the host genome. This work enriched the site-specific recombination system of the producer and facilitated subsequent genetic manipulation. In addition, it is noteworthy that the *gusA* gene can be used as a visual reporter gene for the study of *A. orientalis*.

### Refactoring of regulatory circuits

The biosynthesis of microbial natural products is tightly and complexly regulated by kinds of regulators at multiple levels [31, 32]. Genetic manipulation based on regulatory genes is an effective strategy for the improvement of antibiotic yield [33].

The constructed biosynthetic gene cluster of A82846B contains a pathway-specific regulatory gene, *strR*, which encodes a protein belonging to the StrR family with two functional structural domains. In our previous study, it was confirmed that StrR positively regulates A82846B production [15]. In this study, to further enhance the production of A82846B, we applied the pIJ8660 integrative vector to construct the overexpression plasmids of *strR* driven by the promoter *gapdh* based on the PhiC31 system (Fig. 3D). The plasmid was transformed into the strain AO-4 by conjugal transfer, generating the strain AO-5. The A82846B yield of AO-5 reached 226 mg/L, increased by 146% compared with the wild-type strain (Fig. 3E). These results indicated that the fermentation level of target product was further improved by strengthening the regulatory pathway.

### Strengthening of synthesis pathways

The biosynthesis of microbial natural products necessitates the harmonious operation of multiple gene modules. To enhance the synthesis efficiency of the desired product, it is imperative to concentrate on rate-limiting steps in the synthesis process. Analyzing synthetic pathways and overexpressing the corresponding genes, especially those involved in precursor synthesis and modification, is a conventional approach to ascertaining these bottleneck steps in the synthesis of secondary metabolites.

**Fig. 4:**
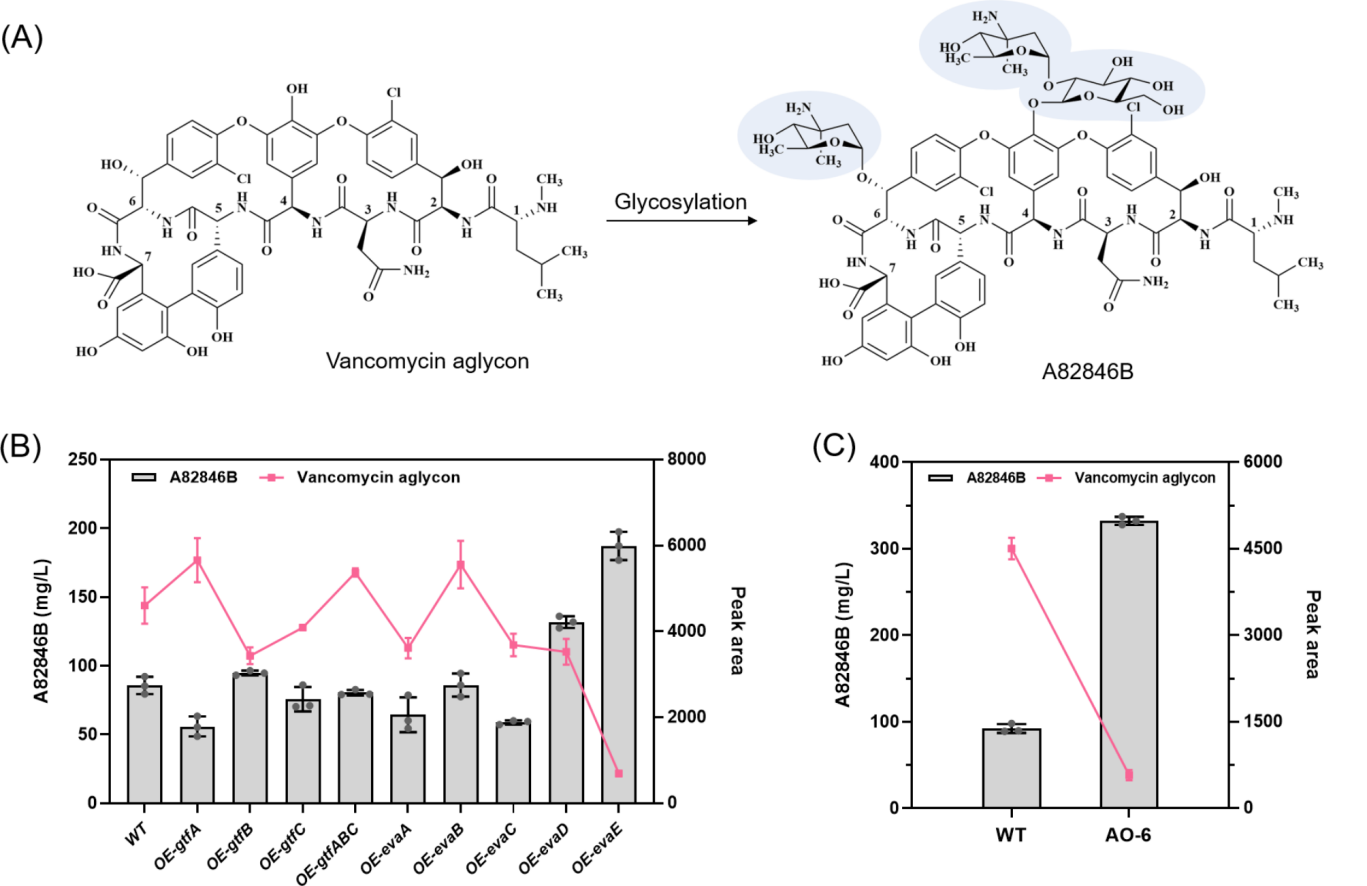
A82846B production and the precursor accumulation (vancomycin aglycone) of the engineered strains and wild-type strain. **(A)** The structure of vancomycin aglycone and A82846B. Glycosyl groups were shown in blue blocks. **(B)** Identification of potential missing bioconversion steps in the A82846B biosynthesis process by overexpressing glycosyl-synthesis and glycosyl-transfer genes. **(C)** Improvement of A82846B production by *evaE* gene overexpression (AO-6) based on multi-step combinatorial strategy. Values are presented as averages ± SD from three independent measurements

Through HPLC analysis of the A82846B fermentation broth, we found that there was a large accumulation of compound X, and the MS spectrum revealed the m/z value of peak X is 1143.09 ([M + H]^+^) (see Additional file: Fig. S3), consistent with the m/z value of vancomycin aglycone according to the reported literature [34]. Vancomycin aglycone was modified by glycosylation to finally generate A82846B, which was an essential intermediate involved in A82846B biosynthesis (Fig. 4A). Because of the large accumulation of vancomycin aglycone, we hypothesized that glycosyl synthesis and glycosyl transfer modules belonging to glycosylation were the major missing bioconversion steps in the A82846B biosynthetic process.

To identify missing bioconversion steps during the process of A82846B biosynthesis, we constructed overexpression plasmids of genes involved in the glycosyl-synthesis module (*evaABCDE*) and glycosyl-transfer module (*gtfABC*), and then transformed these overexpression plasmids into the strain AO-2 via conjugal transfer. These constructed mutant strains (AO-2-1 to AO-2-9) were fermented to further test whether overexpression of glycosylation genes would affect the fermentation level of A82846B and the accumulation of intermediates. As shown in Fig. 4B, the strain with high expression of the glycosyl-synthesis gene *evaE* dramatically increased A82846B yield while the accumulation of vancomycin aglycone was significantly reduced, indicating that the bioconversion catalyzed by EvaE was a missing step in the A82846B biosynthetic process. Afterwards, we introduced the *evaE* overexpression plasmid into the strain AO-5, constructing the mutant strain AO-6. The fermentation level of A82846B of AO-6 was improved to 332 mg/L, an increase of 261% compared with the wild type (Fig. 4C). These results illustrated that the synthetic pathway was rationally optimized to further increase the A82846B production and reduce side products effectively.

### Fermentation optimization

In this study, we first screened the optimal carbon sources and nitrogen sources of the fermentation medium by single-factor experiment. Carbon sources, including maltodextrin, molasses, corn flour and soluble starch were supplemented in the GP medium. Supplementation of maltodextrin showed the most significant improvement of A82846B production compared with other carbon sources (see Additional file: Fig. S4). Peptone, casein hydrolysate, yeast powder, soybean powder, cottonseed meal and meat extract as sole nitrogen sources were added to the culture medium. Among these nitrogen sources, peptone showed a positive fermentation level of A82846B (see Additional file: Fig. S4). Hence, glucose and maltodextrin were selected as the composite carbon source and peptone as the nitrogen source.

Plackett-Burman design was used to screen significant factors involved in A82846B production among seven components, including glucose, peptone, maltodextrin, NaCl, KCl, MgSO_4_ and KH_2_PO_4_. PB design and its experimental responses are shown in Table 2. ANOVA analysis of the data was performed and a statistical model was developed using regression analysis (Table 3). The *p*-value of this model was 0.0060 (< 0.05), suggesting that the regression equation carried statistical significance. The regression coefficient (*R*^2^) was 0.9718, demonstrating that 97.18% of the total variation could be explained by the developed model. Among seven tested factors, the significance order of four significant terms (*p*-values < 0.05) on A82846B production was as follows: peptone > glucose > maltodextrin > MgSO_4_. Therefore, peptone, glucose and maltodextrin were chosen for RSM to determine an optimum value and analyze their interactive effects.

**Table 2:** Design and results of Plackett-Burman experiment

Groups	Factors (g/L)								A82846B (mg/L)
	Glucose	Peptone	Maltodextrin	NaCl		KCl	MgSO_4_	KH_2_PO_4_	
1	20	5	20	1.5	0.5		1.2	0.15	320
2	30	7.5	20	1.5	0.75		1.2	0.1	390
3	30	5	30	1.5	0.75		0.8	0.1	84
4	20	5	20	1	0.5		0.8	0.1	273
5	30	5	30	1.5	0.5		1.2	0.15	132
6	20	7.5	20	1.5	0.75		0.8	0.15	360
7	20	7.5	30	1	0.75		1.2	0.15	369
8	30	7.5	30	1	0.5		0.8	0.15	223
9	20	7.5	30	1.5	0.5		0.8	0.1	402
10	30	5	20	1	0.75		0.8	0.15	159
11	30	7.5	20	1	0.5		1.2	0.1	382
12	20	5	30	1	0.75		1.2	0.1	289

**Table 3:** Analysis of variance for Plackett-Burman experiment

Source	Sum of squares	df	Mean square	F-Value	*P*-value
Model	1.279E + 005	7	18269.70	19.66	0.0060
Glucose	34454.08	1	34454.08	37.08	0.0037
Peptone	62930.08	1	62930.08	67.72	0.0012
Maltodextrin	12352.08	1	12352.08	13.29	0.0219
NaCl	4.08	1	4.08	4.394E-003	0.9503
KCl	546.75	1	546.75	0.59	0.4858
MgSO_4_	12096.75	1	12096.75	13.02	0.0226
KH_2_PO_4_	5504.08	1	5504.08	5.92	0.0717
Residual	3717.00	4	929.25		
Cor Total	1.316E + 005	11			

Note The *p*-values less than 0.05 are significant. *R*^2^ = 97.18%

RSM was a practical tool for optimizing fermentation factors. To find the optimum fermentation concentration of three significant medium compositions, the fermentation conditions were further optimized using BBD with three factors and three levels. Tables 4 and 5 show the experimental responses and the analysis of variance for the response surface model. The experimental data were fitted to a quadratic model using regression analysis. A *P*-value of 0.0053 (< 0.05) in this model indicated that the regression equation was significant. The response equation obtained to predict A82846B production was as follows:

Y = 332.80 + 10.63*A-68.50*B-7.88*C + 3.50*AB-7.25*AC + 36.00*BC-100.78*A^2^-13.53*B^2^-17.77*C^2^

**Table 4:** Design and results of Box-Behnken experiment

Groups	Factors (g/L)			A82846B(mg/L)
	A-Glucose	B-Peptone	C-Maltodextrin	
1	10	10	10	198
2	20	6	10	377
3	20	14	20	298
4	30	10	20	190
5	10	14	15	96
6	30	14	15	147
7	10	6	15	272
8	20	10	15	374
9	20	10	15	404
10	20	10	15	415
11	10	10	20	229
12	20	10	15	370
13	20	6	20	306
14	20	10	15	320
15	30	6	15	334
16	20	14	10	225
17	30	10	10	279

**Table 5:** Analysis of variance for the response surface model

Source	Sum of squares	df	Mean square	F-Value	*P*-value
Model	1.255E + 005	9	13943.27	8.33	0.0053
A	3003.13	1	3003.13	1.8	0.2222
B	34191.12	1	34191.12	20.44	0.0027
C	392	1	392	0.23	0.6431
AB	30.25	1	30.25	0.018	0.8968
AC	3600	1	3600	2.15	0.1858
BC	5184	1	5184	3.1	0.1217
A^2^	61569.92	1	61569.92	36.8	0.0005
B^2^	7939.92	1	7939.92	4.75	0.0658
C^2^	4224.44	1	4224.44	2.53	0.1561
Residual	11710.45	7	1672.92		
Lack of Fit	6231.25	3	2077.08	1.52	0.3394
Pure Error	5479.2	4	1369.8		
Cor Total	1.372E + 005	16			

Note The *p*-values less than 0.05 are significant. *R*^2^ = 91.46%

**Fig. 5:**
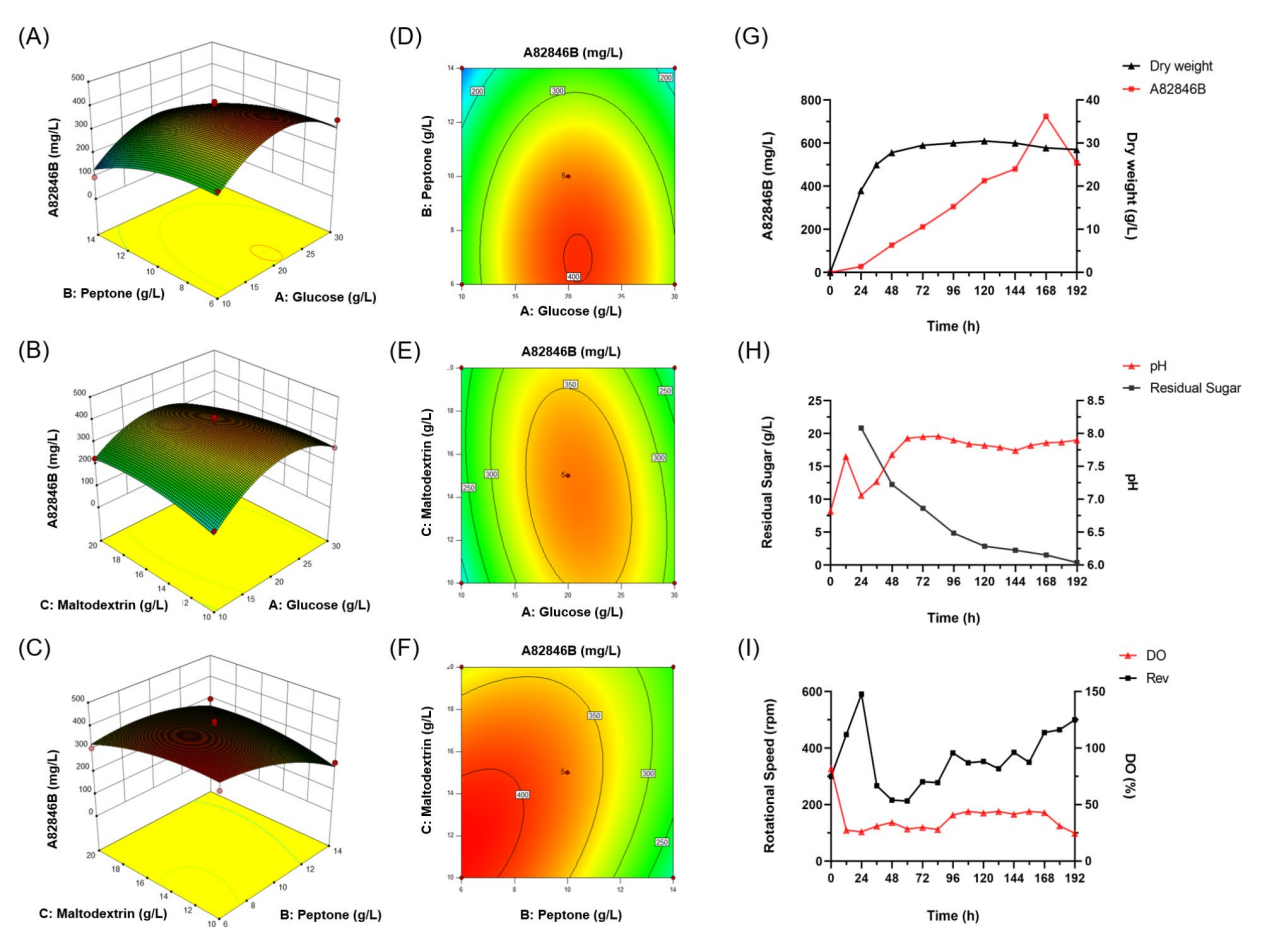
Interaction effects of medium components on A82846B production and the fermentation results of scale-up culture in a 15 L tank. **(A-C)** Effect of glucose, peptone and maltodextrin on A82846B production by 3D response surface plots. **(D-F)** Effect of glucose, peptone and maltodextrin on A82846B production by contour plots. **(G-I)** The curves of A82846B production and dry weight, pH and residual sugar, rotational speed (Rev) and dissolved oxygen (DO)

Three-dimensional response plots and contour plots showed that three variables had interactive effects on the production of A82846B and the convex response surface illustrated that each variable had an optimal value (Fig. 5A-F). Based on the RSM, the optimal values of three tested factors for the maximum A82846B production were predicted as: glucose at 22 g/L, peptone at 6 g/L and maltodextrin at 12 g/L, and A82846B yield was predicted to be 415 mg/L. By confirming through experimentation, the A82846B production reached 401 ± 15 mg/L under the optimal condition (GPM medium), which was similar to the predicted maximum value.

To further optimize the fermentation process, fermentation conditions including temperature, initial pH, seed age, inoculating volume and medium volume were tested for the effect on A82846B production by single-factor experiment. The ideal conditions were obtained: temperature at 30℃, initial pH at 6.5, seed age at 72 h, inoculating volume at 2% and medium volume at 25 mL (see Additional file: Fig. S4). Finally, under optimized culture medium and conditions, the fermentation level of A82846B was increased to 436 mg/L, showing a significant improvement of 4.74 folds, which was the highest A82846B production without homologous impurities at the shaker fermentation level reported thus far.

## Scale-up fermentation

In industrial production, it is necessary to scale up the fermentation process to explore the most suitable fermentation techniques at different production scales for meeting the requirements of commercial production. Therefore, based on the fermentation optimization at the shake flask level, this study conducted the scale-up cultivation in a 15 L fermenter.

At the initial stage of scale-up fermentation, multiple trials were conducted to preliminarily explore the fermentation process and fermenter parameter settings. During this process, various fermentation issues encountered were analyzed, and a series of improvement measures were carried out. For example, calcium carbonate was added to the fermentation medium to stabilize the significant pH changes during the fermentation process and increase the adhesion of the microbial cells. Besides, the medium formula of seed culture, inoculation time, seed culture mode and inoculation volume were optimized as well. Moreover, we further improved the content of carbon and nitrogen sources of the fermentation medium (an increase of 0.3% glucose, 1.2% peptone and 3.8% maltodextrin), designing the GPM-2 medium with the hope that the increase in nutrient components would be conducive to a further increase in A82846B yield. The fermentation results of scale-up cultivation in a 15 L fermenter are shown in Fig. 5G-I.

The fermentation results showed that A82846B began to accumulate from 24 h, entering a rapid accumulation phase in the later stages of fermentation, with the yield reaching its maximum value of 725 mg/L at 168 h. Observation of the microbial growth curve revealed that between 0 and 48 h, the microorganisms were in a rapid growth phase, during which the rate of sugar consumption was also fast, indicating that the microorganisms extensively utilized nutrients for primary metabolic activities in the early stages of fermentation. After 48 h, the growth of the microorganisms stabilized, and product accumulation increased, indicating that the microorganisms had entered the secondary metabolic activity stage. From the pH change curve, it can be seen that there were no significant fluctuations in pH throughout the fermentation process, due to the buffering action of phosphates and calcium carbonate added to the medium. The reducing sugar content curve showed that the sugars in the medium were exhausted only after 192 h of fermentation, indicating that there was an ample supply of carbon sources throughout the fermentation process. The optimized medium formulation was reasonable and could effectively support the production of A82846B. Additionally, it was worth noting that the optimization of process conditions had a great effect on increasing production. In this study, we conducted a preliminary exploration of the process conditions, and more work can be done in subsequent research.

**Fig. 6:**
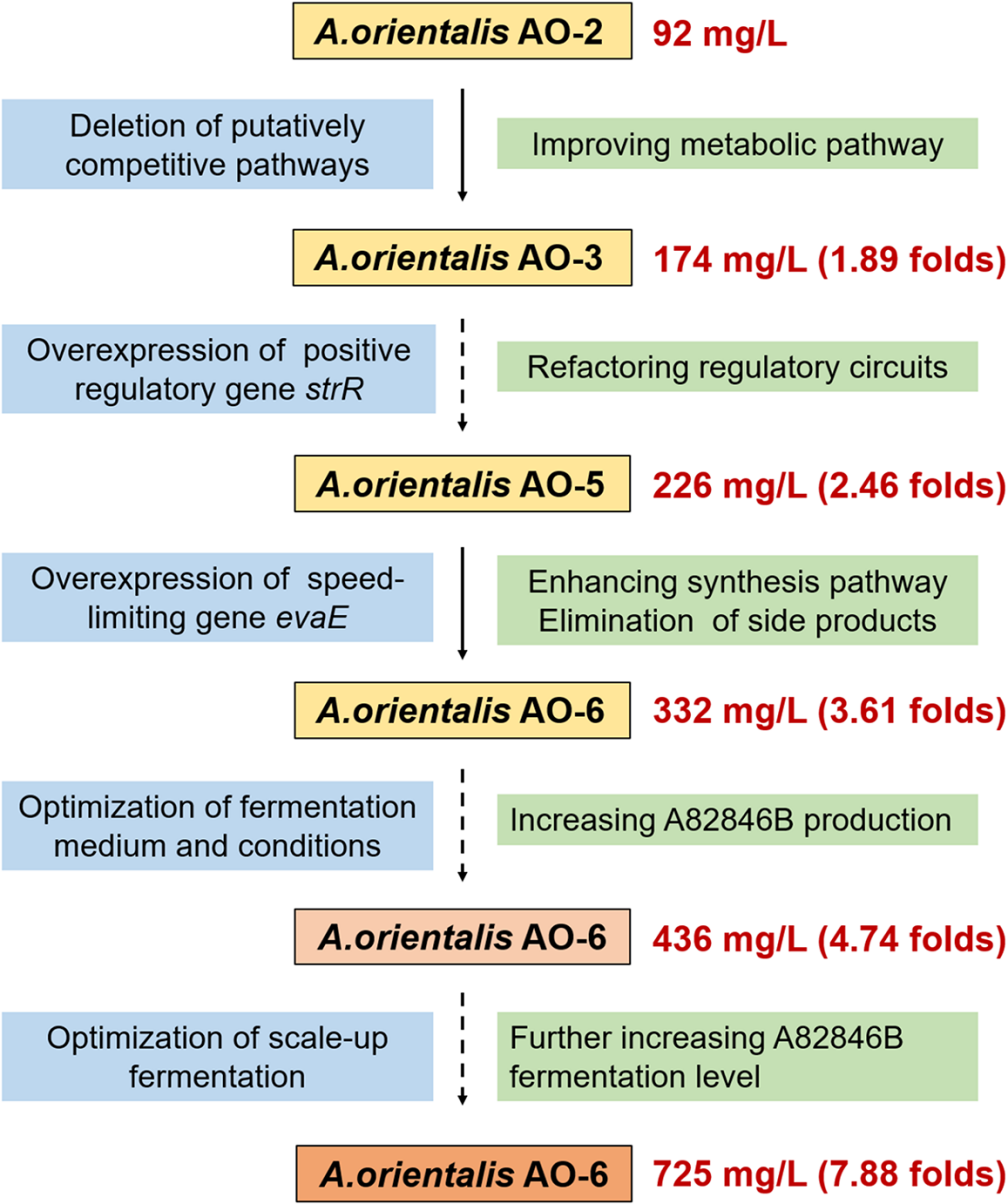
Diagram summarizing the strategies used to improve the fermentation level of A82846B in *A. orientalis*. The red data represented the A82846B yields of each strain and the corresponding fold change in comparison to the parental strain AO-2

**Fig. 7:**
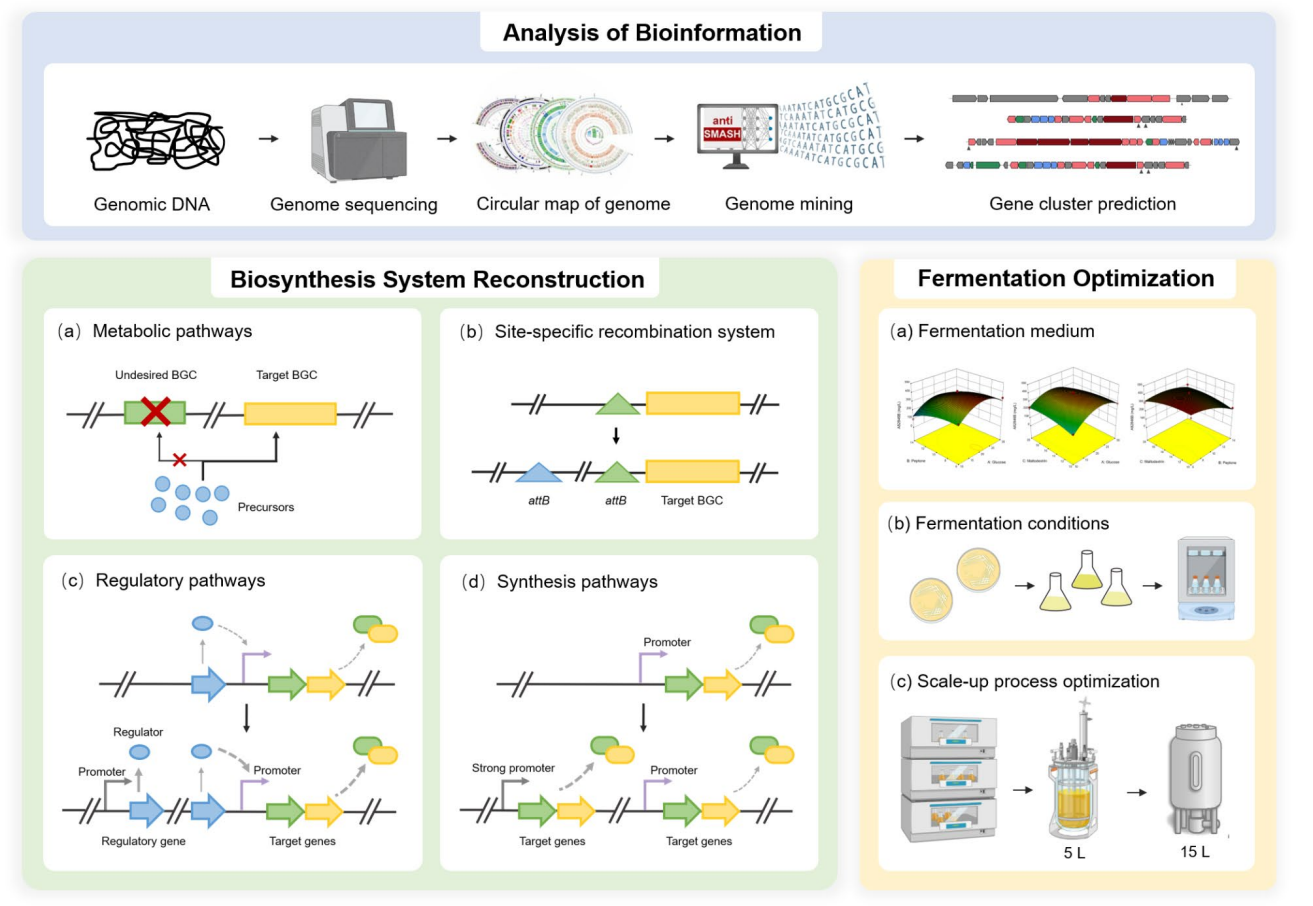
The multi-step combinatorial strategy used to improve the production of A82846B

In summary, based on the results of shake flask fermentation optimization and multiple experiments in fermenters, taking into account factors such as equipment and environment, we explored the fermentation process for scaling up the production of A82846B. Ultimately, using the optimized fermentation process, the high-yielding strain AO-6 achieved an A82846B yield of 725 mg/L in a 15 L fermenter, which is the highest reported yield of A82846B without the generation of homologous impurities.

## Conclusions

In this study, we report the achievement of high-quality and high-efficiency production of Oritavancin direct precursor A82846B through combinatorial strategies involving constructing an efficient biosynthetic system and fermentation optimization (Fig. 6). The fermentation level of A82846B-producing strain *A. orientalis* AO-6 was reached 725 mg/L, showing a significant improvement of 7.88 folds. To the best of our knowledge, this is the highest A82846B yield without the generation of homologous impurities ever reported. The multi-step combinatorial strategy can be widely used in the hyper-production of other valuable natural products derived from microorganisms (Fig. 7).

### Electronic supplementary material

Below is the link to the electronic supplementary material.

Supplementary Material 1

## Data Availability

No datasets were generated or analysed during the current study.

## Abbreviations

ABSSSI: Acute bacterial skin and skin structure infections
PKS: Polyketide synthase
NRPS: Non‑ribosomal peptide synthase
RT‑PCR: Reverse transcription polymerase chain reaction
qRT‑PCR: Quantitative real‑time polymerase chain reaction
HPLC: High‑performance liquid chromatography
UV: Ultraviolet light; PBD: Plackett-Burman design
BBD: Box-Behnken design
RSM: Response surface methodology
ANOVA: Analysis of variance
