# Supplementary material for: Rational construction of a high-quality and high-efficiency biosynthetic system and fermentation optimization for A82846B based on combinatorial strategies in Amycolatopsis orientalis
Source: Microb Cell Fact. 2024 Jun 28;23:186. doi: 10.1186/s12934-024-02464-4 (PMC11212272; doi:10.1186/s12934-024-02464-4)
Supplement: Supplementary file 1 — Supplementary Material 1 [file 12934_2024_2464_MOESM1_ESM.docx]

**Supplementary material** for

Rational construction of a high-quality and high-efficiency biosynthetic system and fermentation optimization for A82846B based on combinatorial strategies in *Amycolatopsis orientalis*

**Summary**

The file includes 4 diagrams and 2 tables.

**Fig. S1** is used to point to Section “**Deletion of putatively competitive gene cluster**”.

**Fig. S2** is used to point to Section “**Deletion of putatively competitive gene cluster**”.

**Fig. S3** is used to point to Section “**Strengthening of synthesis pathways**”.

**Fig. S4** is used to point to Section “**Fermentation optimization**”.

**Table S1** and **Table S2** are used to point to Section “**Strains, plasmids, and primers**”.


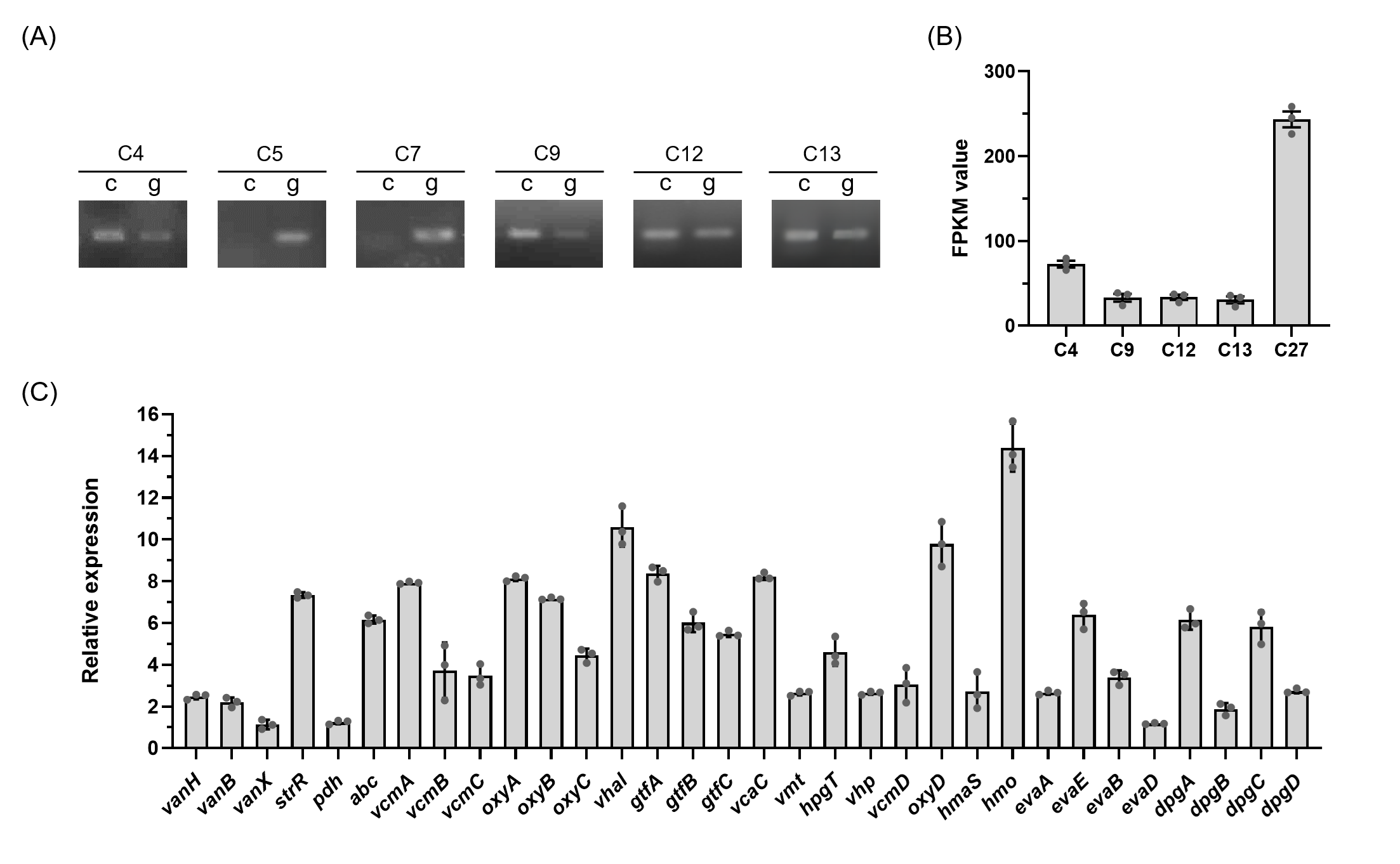


**Fig. S1.** Transcription analysis involved in deleting potential competitive pathways. **(A)** Analysis of gene clusters expression in strain AO-2 by RT-PCR. Complementary DNA (c); Genome DNA (g). **(B)** RNA-seq FPKM value of four transcribed NRPS/NRPS-PKS gene clusters in strain AO-2. C7: A82846B gene cluster. **(C)** Effect on the transcriptional level of A82846B cluster genes after deleting key genes in C4.


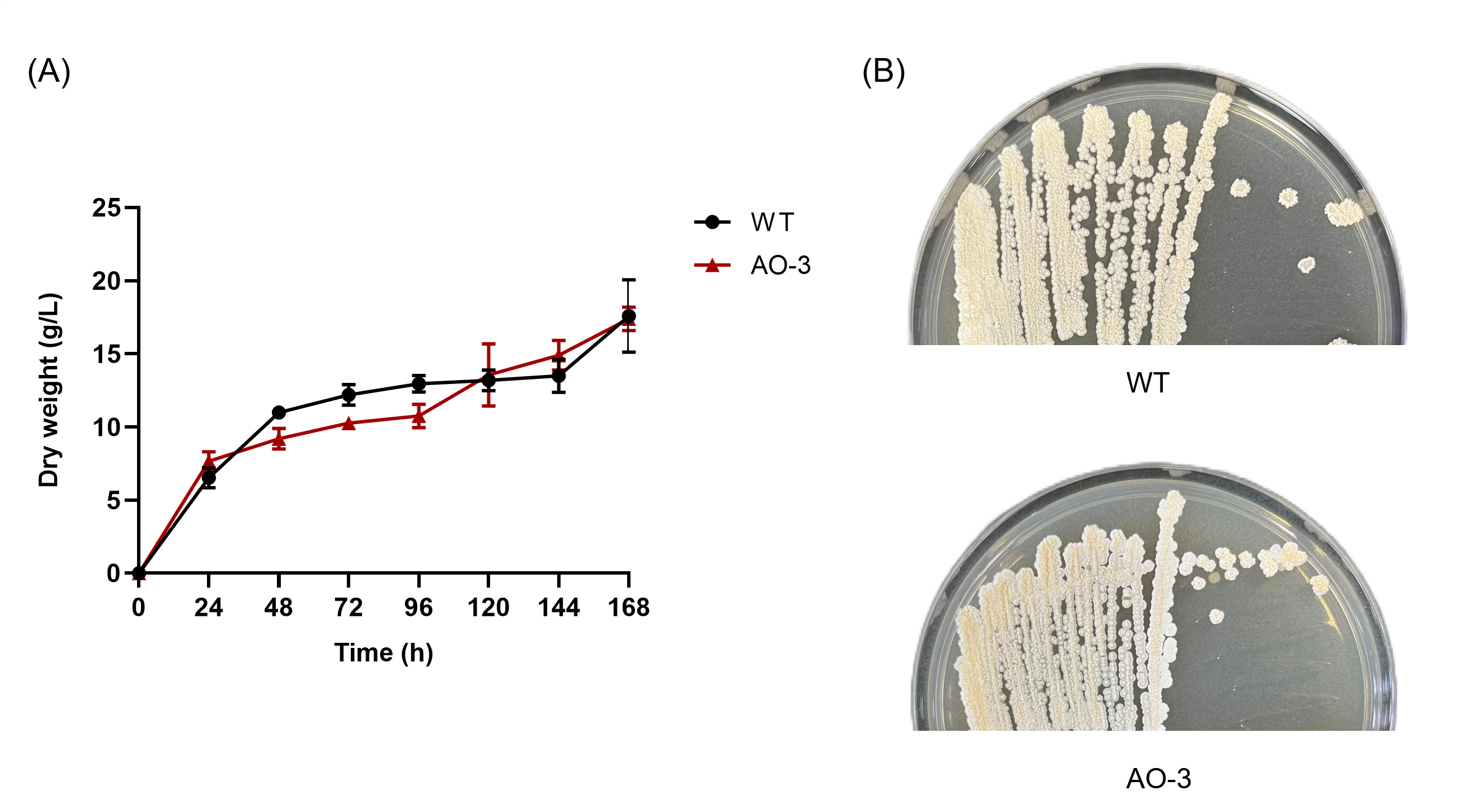


**Fig. S2.** Effect of deleting competitive gene cluster on physiological parameters of strains. **(A)** Cell growth. **(B)** Colony morphology and colour.


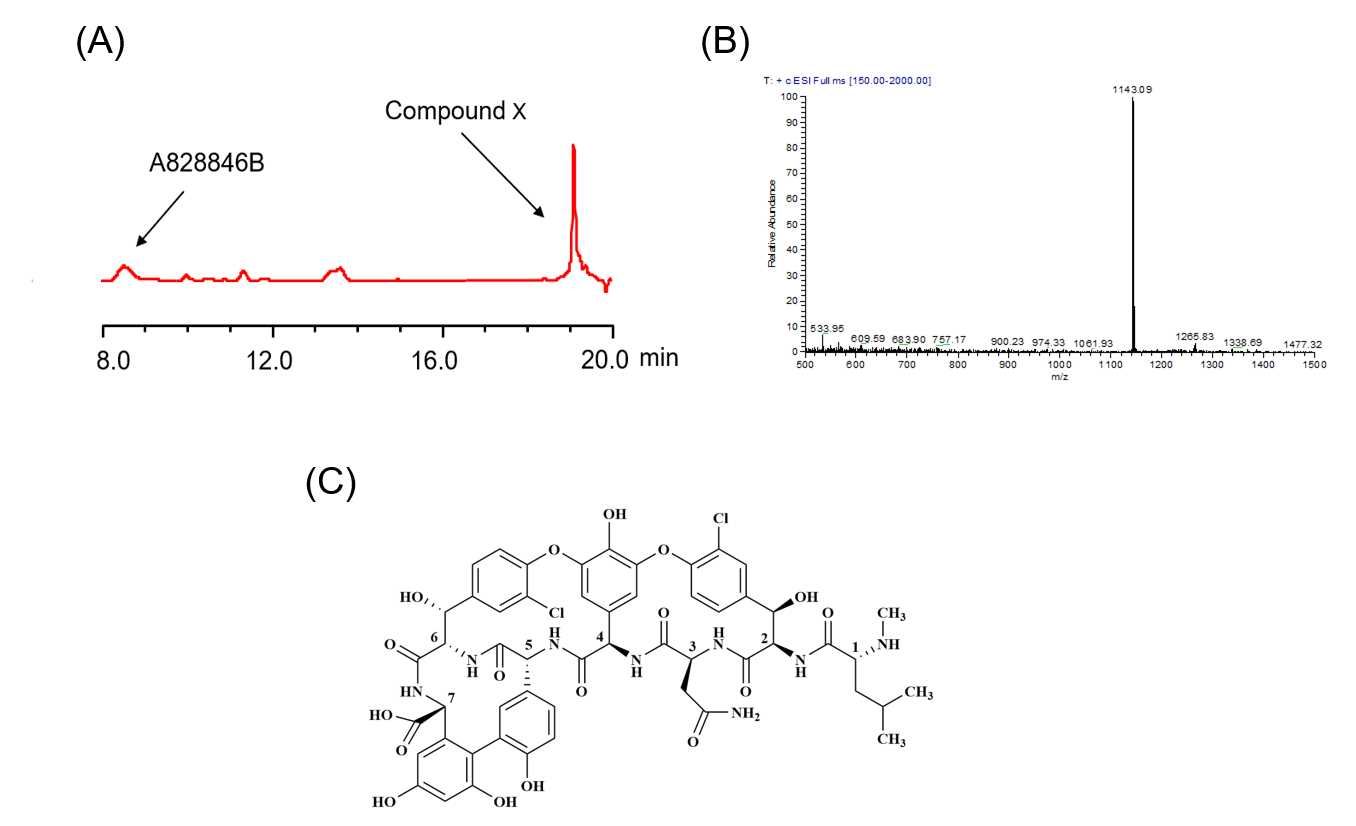


**Fig. S3.** Analysis of the fermentation culture of *A. orientalis* AO-2. **(A)** HPLC analysis of the fermentation broth of *A. orientalis* AO-2 and Compound X were accumulated. **(B)** MS analysis of the putative Vancomycin aglycone accumulated in AO-2 fermentation culture. [M+H] ^+^= 1143.09. **(C)** The structure of Vancomycin aglycone.


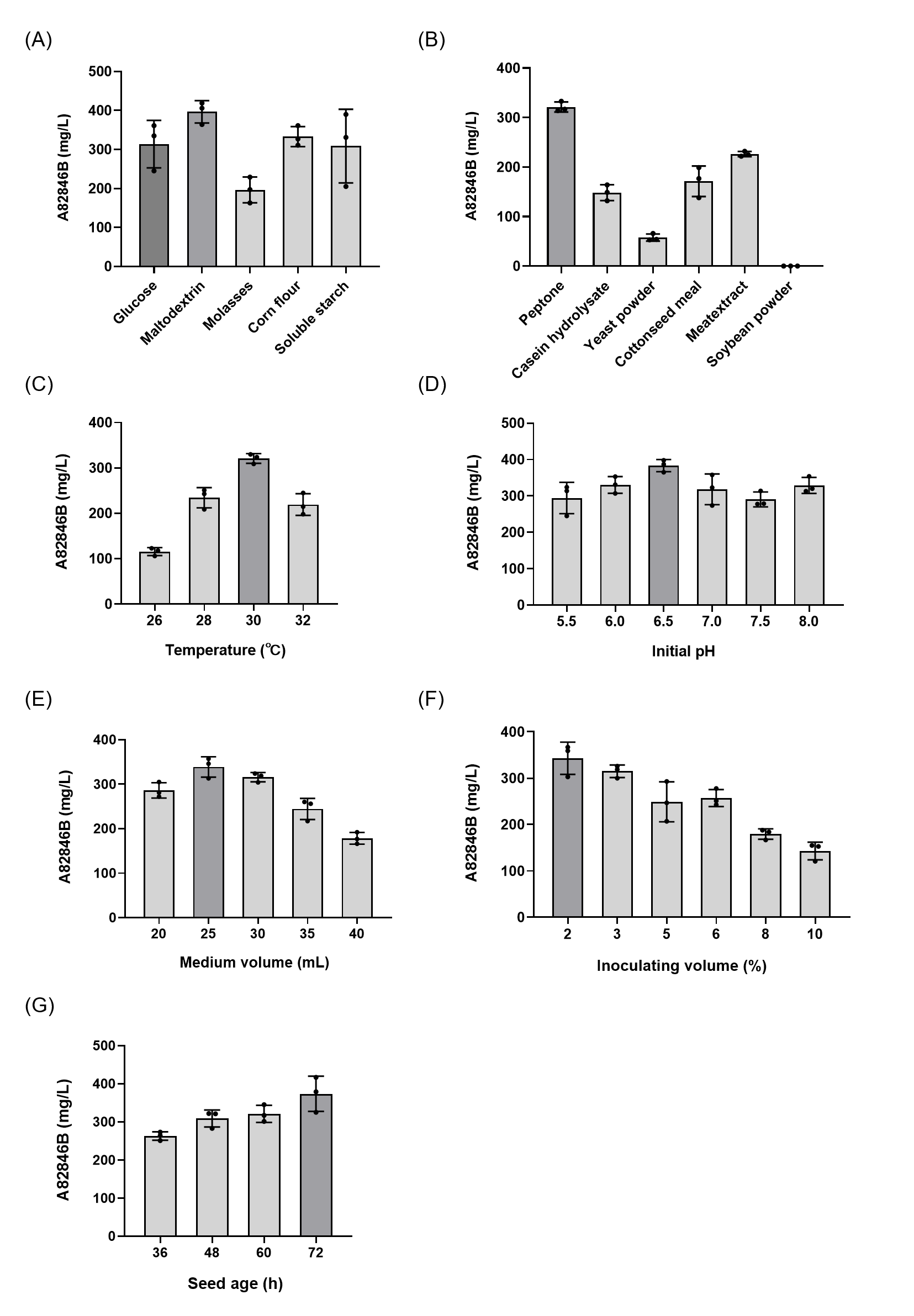


**Fig. S4** Effect of medium composition and conditions on A82846B production. **(A)** Different carbon sources. **(B)** Different nitrogen sources. **(C)** Fermentation temperature. **(D)** Initial pH value. **(E)** Medium volume. **(F)** Inoculating volume. **(G)** Seed age.

**Table S1.** Strains and plasmids used in this study

| **Strains or plasmids** | **Description** | **Reference** |
| --- | --- | --- |
| **Strains** |  |  |
| *A. orientalis* AO-2 | Wild type A82846B producer | [1] |
| *A. orientalis* AO-3 | Blocking putative competitive pathways (C4), derived from AO-2 | This study |
| *A. orientalis* AO-4 | Integrated with ΦC31-*attB* site, derived from AO-3 | This study |
| *A. orientalis* AO-4-*gusA* | *A. orientalis* AO-4 containing pIJ8660-*aadA/neo-gusA*, Apr^R^ | This study |
| *A. orientalis* AO-5 | Over-expressing *strR*, derived from *A. orientalis* AO-4, Kan^R^ | This study |
| *A. orientalis* AO-6 | Over-expressing *evaE*, derived from *A. orientalis* AO-5, Kan^R^ /Apr^R^ | This study |
| *A. orientalis* AO-2-1 | *A. orientalis* AO-2 containing pSOK804-*gtfA*, Apr^R^ | This study |
| *A. orientalis* AO-2-2 | *A. orientalis* AO-2 containing pSOK804-*gtfB*, Apr^R^ | This study |
| *A. orientalis* AO-2-3 | *A. orientalis* AO-2 containing pSOK804-*gtfC*, Apr^R^ | This study |
| *A. orientalis* AO-2-4 | *A. orientalis* AO-2 containing pSOK804-*gtfABC*, Apr^R^ | This study |
| *A. orientalis* AO-2-5 | *A. orientalis* AO-2 containing pSOK804-*evaA*, Apr^R^ | This study |
| *A. orientalis* AO-2-6 | *A. orientalis* AO-2 containing pSOK804-*evaB*, Apr^R^ | This study |
| *A. orientalis* AO-2-7 | *A. orientalis* AO-2 containing pSOK804-*evaC*, Apr^R^ | This study |
| *A. orientalis* AO-2-8 | *A. orientalis* AO-2 containing pSOK804-*evaD*, Apr^R^ | This study |
| *A. orientalis* AO-2-9 | *A. orientalis* AO-2 containing pSOK804-*evaE*, Apr^R^ | This study |
| *E. coli* DH5α | General cloning strain | Novagen, China |
| *E. coli* ET12567(pUZ8002) | Donor strain for *E. coli*-Actinomyces conjugation | FengBio, China |
| **Plasmids** |  |  |
| pSET153 | Suicide plasmid derived from pSET152 | [1] |
| pSET153-ΔC4 | For deletion of core biosynthetic genes in cluster C4 | This study |
| pKCCas12a | CRISPR plasmid, Cas12a under control of *tipA* and crRNA under control of *kasO*p* | [1] |
| pKCCas12a-ΦC31 | For integration of the ΦC31-*attB* site | This study |
| pIJ8660-*aadA/neo* | Integrative plasmid bearing spe^R^/kan^R^, *attP*, intΦC31 | [2] |
| pIJ8660-*aadA/neo-gusA* | Integrative plasmid bearing spe^R^/kan^R^, *attP*, intΦC31, with *gusA* reporter system | This study |
| pIJ8660-*aadA/neo-strR* | For overexpression of the regulatory gene *strR* | This study |
| pSOK804 | Integrative plasmid bearing apr^R^, *attP*, intVWB | [3] |
| pSOK804G | Integrative plasmid bearing apr^R^, *attP*, intVWB, promoter *gapdh* | This study |
| pSOK804G-*gtfA* | Plasmid used for overexpression of the glycosyl transfer gene *gtfA* | This study |
| pSOK804G-*gtfB* | Plasmid used for overexpression of the glycosyl transfer gene *gtfB* | This study |
| pSOK804G-*gtfC* | Plasmid used for overexpression of the glycosyl transfer gene *gtfC* | This study |
| pSOK804G-*gtfABC* | Plasmid used for overexpression of the glycosyl transfer gene *gtfABC* | This study |
| pSOK804G-*evaA* | Plasmid used for overexpression of the glycosyl synthesis gene *evaA* | This study |
| pSOK804G-*evaB* | Plasmid used for overexpression of the glycosyl synthesis gene *evaB* | This study |
| pSOK804G-*evaC* | Plasmid used for overexpression of the glycosyl synthesis gene *evaC* | This study |
| pSOK804G-*evaD* | Plasmid used for overexpression of the glycosyl synthesis gene *evaD* | This study |
| pSOK804G-*evaE* | Plasmid used for overexpression of the glycosyl synthesis gene *evaE* | This study |

Note: Apr^R^ apramycin resistance, Kan^R^ kanamycin resistance, Spe^R^ spectinomycin resistance.

**Table S2.** Primers used in this study

| **Pairs** | **Primers** | | **Sequence (5’-3’)** | **Description** |
| --- | --- | --- | --- | --- |
|  | C4-F | | gctttccctgctggcac | RT-PCR primers for cluster C4 |
|  | C4-R | | cacagtggagcgtcgagg |  |
|  | C5-F | | gtcgaaacgctctacggcg | RT-PCR primers for cluster C5 |
|  | C5-R | | gtcgaccgcacgggtcc |  |
|  | C7-F | | cggagtaccaccgggtg | RT-PCR primers for cluster C7 |
|  | C7-R | | gaactccccgccgagttc |  |
|  | C9-F | | ctcatggggcagatcgcg | RT-PCR primers for cluster C9 |
|  | C9-R | | caggccgtcgaacacgt |  |
|  | C12-F | | gttcctcgtcagcgaggc | RT-PCR primers for cluster C12 |
|  | C12-R | | ggcgaggtcgttcaggaac |  |
|  | C13-F | | ggtggtgaacccgtccag | RT-PCR primers for cluster C13 |
|  | C13-R | | caggtcttcggcgaggtg |  |
|  | C4-LA-F | | ctagagtcgacctgcagccccgtgagctggccgaaaccga | Amplification of 2.5 kb left homologous arm of C4 |
|  | C4-LA-R | | cgatggcgaccgtggccgcaggacgaagtcgccgagcg |  |
|  | C4-RA-F | | gcggccacggtcgccatcggggt | Amplification of 2.5 kb right homologous arm of C4 |
|  | C4-RA-R | | ctcttccacctgctgaaagcttcggcgtctccgccttcttcgt |  |
|  | ΔC4-F | | cgacgtcggggagctcgtcacgc | Identification of the deletion of core biosynthetic genes in cluster C4 |
|  | ΔC4-R | | ggcagttcgatgccggttgtccc |  |
|  | ΦC31-attB-F | | acgttgcattcctaccaacggtgcgggtgccagggcgt | Amplification of ΦC31-*attB* site |
|  | ΦC31-attB-R | | tggtcgatctgcaccgcgtacgcgcccggggagcccaa |  |
|  | ΦC31-LA-F | | cacgccgcagagctgactagttgtgcatcacctccagctg | Amplification of 2.5 kb left homologous arm for ΦC31-*attB* insertion area |
|  | ΦC31-LA-R | | acgccctggcacccgcaccgttggtaggaatgcaacgtgagac |  |
|  | ΦC31-RA-F | | tgggctccccgggcgcgtacgcggtgcagatcgaccac | Amplification of 2.5 kb right homologous arm for ΦC31-*attB* insertion area |
|  | ΦC31-RA-R | | atatcgacgctctagagctagcagcagtttgtgcagcagc |  |
|  | Cas12a-ΦC31-spacer-F | | gaactcctggtagatggacatatgtgtccgctcccttct | Amplification of the spacer of crRNA for ΦC31-*attB* insertion area, under the promoter *kasO*p* |
|  | Cas12a-ΦC31-spacer-R | | tggaggtgatgcacaactagttctgtcctttgaggacgatcaggatctacaacagtagaaatt |  |
|  | In-ΦC31-F | | ctccagccgtgaagcga | Identification of the insertion of ΦC31-*attB* site |
|  | In-ΦC31-R | | ggctgttccccacggtca |  |
|  | strR-F | | aaactttagatcctcgagatctgctgctccttcggtcgga | Amplification of *strR*, under the promoter *gapdh* |
|  | strR-R | | catcttgttcaatcatcatatgttacgcgatcgctcccgt |  |
|  | gtfA-F | | gaaaggggatacgcatgcgcgtgttgattacggg | Amplification of *gtfA* |
|  | gtfA-R | | aacagctatgacatgattactcaggcgggaacagtcggct |  |
|  | gtfB-F | | tgaaaggggatacgcggtaccatgcgtgtgctgttggcgacgtg | Amplification of *gtfB* |
|  | gtfB-R | | tatgacatgattacgaattcttacgcggaaacagtcggct |  |
|  | gtfC-F | | gaaaggggatacgcggtaccatgcgtgtgttgttgtcgacgg | Amplification of *gtfC* |
|  | gtfC-R | | gaaacagctatgacatgattacttacgcgagaacagccgac |  |
|  | gtfABC-F | | gaaaggggatacgcatgcgcgtgttgattacggg | Amplification of *gtfABC* |
|  | gtfABC-R | | gaaacagctatgacatgattacttacgcgagaacagccgac |  |
|  | evaA-F | | tgaaaggggatacgcggtaccatgtcgtccttcgtcgttcc | Amplification of *evaA* |
|  | evaA-R | | tatgacatgattacgaattctcatgcacctccccgaggctggg |  |
|  | evaB-F | | gaaaggggatacgcggtaccatgaccacgcgtgtatggga | Amplification of *evaB* |
|  | evaB-R | | tatgacatgattacgaattctcagagggtcgacagcact |  |
|  | evaC-F | | ctgaaaggggatacgcggtaccatgtcgaccacttcccgg | Amplification of *evaC* |
|  | evaC-R | | tatgacatgattacgaattcgctcacgtgcacgttcg |  |
|  | evaD-F | | tgaaaggggatacgcggtaccgtgcaagcacgcaaactcg | Amplification of *evaD* |
|  | evaD-R | | tatgacatgattacgaattctcaggtcgagacggggacc |  |
|  | evaE-F | | gaaaggggatacgcggtaccatgaagctgatcaccgtgct | Amplification of *evaE* |
|  | evaE-R | | tatgacatgattacgaattctcatgcgcgagcctttccatg |  |
| **qPCR** | |  |  |  |
|  | vanH-qPCR-F | | ggacggcgcgtacatcatc | For amplifying partial sequence of *vanH* |
|  | vanH -qPCR-R | | ttccggcagtcggcgta |  |
|  | vanB-qPCR-F | | tcgttcggcgtcagcaag | For amplifying partial sequence of *vanB* |
|  | vanB -qPCR-R | | cgccgaggatcgagcag |  |
|  | vanX-qPCR-F | | cctgacgctctaccgcctg | For amplifying partial sequence of *vanX* |
|  | vanX -qPCR-R | | ttccatgatcgcgcacagg |  |
|  | strR-qPCR-F | | cttccgcccattctcgtgc | For amplifying partial sequence of *strR* |
|  | strR-qPCR-R | | gatgaaccgtgcctggatttt |  |
|  | pdh-qPCR-F | | gcatctcgtgggtgagcgactg | For amplifying partial sequence of *pdh* |
|  | pdh-qPCR-R | | gatggcccggcacataggaa |  |
|  | abc-qPCR-F | | tgctcgggcggatgttc | For amplifying partial sequence of *abc* |
|  | abc-qPCR-R | | tggatgagcggcttgagg |  |
|  | vcmA-qPCR-F | | cgtccacggggatgtcac | For amplifying partial sequence of *vcmA* |
|  | vcmA-qPCR-R | | caggatgtggtggatgacgac |  |
|  | vcmB-qPCR-F | | gcgtggaggggatggtg | For amplifying partial sequence of *vcmB* |
|  | vcmB-qPCR r | | cggactgccgcttctgc |  |
|  | vcmC-qPCR-F | | cgtcaaccgggtgcaagaa | For amplifying partial sequence of *vcmC* |
|  | vcmC-qPCR-R | | ccgcaaacccacctggaaca |  |
|  | oxyA-qPCR-F | | gagggccgcatgtccgtgat | For amplifying partial sequence of *oxyA* |
|  | oxyA-qPCR-R | | cgggtcgtactggttgaggaagc |  |
|  | oxyB-qPCR-F | | gccagggaacgcaaggaa | For amplifying partial sequence of *oxyB* |
|  | oxyB-qPCR-R | | cgatcatgccggagatgttgt |  |
|  | oxyC-qPCR-F | | cggctcgccatcaagga | For amplifying partial sequence of *oxyC* |
|  | oxyC-qPCR-R | | agcggcaccgacgcagtaa |  |
|  | vhal-qPCR-F | | aagaacgccaagagcaaggg | For amplifying partial sequence of *vhal* |
|  | vhal-qPCR-R | | gcgaggctgcggaagaa |  |
|  | gtfA-qPCR-F | | gtcggcgaagtgaaccttcagg | For amplifying partial sequence of *gtfA* |
|  | gtfA-qPCR-R | | cctgctccaccacgttgtcca |  |
|  | gtfB-qPCR-F | | gcgcagtgggagcggaacaa | For amplifying partial sequence of *gtfB* |
|  | gtfB-qPCR-R | | cggtgtagccgaaggtgaagat |  |
|  | gtfC-qPCR-F | | cgtggatcctgccggacg | For amplifying partial sequence of *gtfC* |
|  | gtfC-qPCR-R | | cggatcgcctcgatggcc |  |
|  | vcaC-qPCR-F | | cggcgacgagaagaagga | For amplifying partial sequence of *vcaC* |
|  | vcaC-qPCR-R | | gaaccggacgagtgataggg |  |
|  | vmt-qPCR-F | | gtggtcatcagctcgggttcg | For amplifying partial sequence of *vmt* |
|  | vmt-qPCR-R | | cgggcttcgtcaattccag |  |
|  | hpgT-qPCR-F | | gcccgtacgcgttgtctt | For amplifying partial sequence of *hpgT* |
|  | hpgT-qPCR-R | | gccgacgaacagggcatg |  |
|  | vhp-qPCR-F | | tccgggccatgcacaacc | For amplifying partial sequence of *vhp* |
|  | vhp-qPCR-R | | ctcgaggcggtcgggcaa |  |
|  | vcmD-qPCR-F | | cccaggcggtggtgacc | For amplifying partial sequence of *vcmD* |
|  | vcmD-qPCR-R | | gctgacgaccacgtcgg |  |
|  | oxyD-qPCR-F | | tgctgatcggcggcaac | For amplifying partial sequence of *oxyD* |
|  | oxyD-qPCR-R | | gaggtccagcgcagcac |  |
|  | hmaS-qPCR-F | | cacatcgtggtcggcgc | For amplifying partial sequence of *hmaS* |
|  | hmaS-qPCR-R | | gacccgtggtgctccttg |  |
|  | hmo-qPCR-F | | aggaatcctcgcggtcgag | For amplifying partial sequence of *hmo* |
|  | hmo-qPCR-R | | gccggaaagggtggcg |  |
|  | evaA-qPCR-F | | gtcgtgtctgccctaccagg | For amplifying partial sequence of *evaA* |
|  | evaA -qPCR-R | | gccttgcttccagccgcac |  |
|  | evaE -qPCR-F | | caaatggccaggtgcgcg | For amplifying partial sequence of *evaE* |
|  | evaE -qPCR-R | | tcgccccagatggtgagtg |  |
|  | evaB -qPCR-F | | acgaggtccaggcggaaatc | For amplifying partial sequence of *evaB* |
|  | evaB -qPCR-R | | tgctcgttgccgggca |  |
|  | evaD -qPCR-F | | taccttccggtcggcgtc | For amplifying partial sequence of *evaD* |
|  | evaD -qPCR-R | | cgatgtcgatgggcaggcc |  |
|  | dpgA -qPCR-F | | gtgctgtgtcacctcgacc | For amplifying partial sequence of *dpgA* |
|  | dpgA -qPCR-R | | cgcgaccacgttgagcg |  |
|  | dpgB -qPCR-F | | cgatgctgcgcttcgacg | For amplifying partial sequence of *dpgB* |
|  | dpgB -qPCR-R | | cttcgcccagccctcgg |  |
|  | dpgC -qPCR-F | | cgacatcccgctggtgg | For amplifying partial sequence of *dpgC* |
|  | dpgC -qPCR-R | | gccgatggcgaacgaatc |  |
|  | dpgD -qPCR-F | | cgtcgacgttcggcagc | For amplifying partial sequence of *dpgD* |
|  | dpgD -qPCR-R | | gcgacgacgatgtcgca |  |
|  | hrdb-qRCR-F | | atggcgttcctggacctga | For amplifying partial sequence of *hrdb* |
|  | hrdb-qRCR-R | | cgtggagaacttgtagcccttg |  |

**Supplementary Reference**

1. Qian H, Wei W, Chen X-A, Mo X-T, Ge M, Zhao Q-W, et al. Strategy for producing the high-quality glycopeptide antibiotic A82846B in *Amycolatopsis orientalis* based on the CRISPR-Cas12a system. ACS Synth Biol. 2021;10:3009-16.

2. Luo S, Chen X-A, Mao X-M, Li Y-Q. Transposon-based identification of a negative regulator for the antibiotic hyper-production in *Streptomyces*. Appl Microbiol Biotechnol. 2018;102:6581-92.

3. Sekurova ON, Brautaset T, Sletta H, Borgos SEF, Jakobsen ØM, Ellingsen TE, et al. In vivo analysis of the regulatory genes in the nystatin biosynthetic gene cluster of *Streptomyces noursei* ATCC 11455 reveals their differential control over antibiotic biosynthesis. J Bacteriol. 2004;186:1345-1354.
